# Ketamine Reduces Inflammation Pathways in the Hypothalamus and Hippocampus Following Transient Hypoxia in the Late-Gestation Fetal Sheep

**DOI:** 10.3389/fphys.2018.01858

**Published:** 2019-01-07

**Authors:** Eileen I. Chang, Miguel A. Zarate, Thomas J. Arndt, Elaine M. Richards, Maria B. Rabaglino, Maureen Keller-Wood, Charles E. Wood

**Affiliations:** ^1^Department of Physiology and Functional Genomics, University of Florida College of Medicine, Gainesville, FL, United States; ^2^Department of Pharmacodynamics, University of Florida College of Pharmacy, Gainesville, FL, United States; ^3^CEPROCOR, National Scientific and Technical Research Council (CONICET), Córdoba, Argentina

**Keywords:** fetal hypoxia, ketamine, fetal brain, inflammation, transcriptomics

## Abstract

The physiological response to hypoxia in the fetus has been extensively studied with regard to redistribution of fetal combined ventricular output and sparing of oxygen delivery to fetal brain and heart. Previously, we have shown that the fetal brain is capable of mounting changes in gene expression that are consistent with tissue inflammation. The present study was designed to use transcriptomics and systems biology modeling to test the hypothesis that ketamine reduces or prevents the upregulation of inflammation-related pathways in hypothalamus and hippocampus after transient hypoxic hypoxia. Chronically catheterized fetal sheep (122 ± 5 days gestation) were subjected to 30 min hypoxia (relative reduction in P_a_O_2_∼50%) caused by infusion of nitrogen into the inspired gas of the pregnant ewe. RNA was isolated from fetal hypothalamus and hippocampus collected 24 h after hypoxia, and was analyzed for gene expression using the Agilent 15.5 k ovine microarray. Ketamine, injected 10 min prior to hypoxia, reduced the cerebral immune response activation to the hypoxia in both brain regions. Genes both upregulated by hypoxia and downregulated by ketamine after hypoxia were significantly associated with gene ontology terms and KEGG pathways that are, themselves, associated with the tissue response to exposure to bacteria. We conclude that the results are consistent with interruption of the cellular response to bacteria by ketamine.

## Introduction

Transient fetal hypoxia is a common homeostatic disturbance during fetal life. Any compromise of maternal oxygen supply, for example, high altitude or maternal hypoventilation secondary to any cause, decreases partial pressure of oxygen in the fetal blood (Assali et al., 1962; Makowski et al., 1968). Physiologists commonly view the fetal response to hypoxia through the lens of homeostatic cardiovascular responses that spare brain and heart viability (Dawes et al., 1969; Cohn et al., 1974). Cardiovascular and neuroendocrine reflex responses to hypoxia redirect blood flow such that a larger proportion of the combined ventricular output flows toward the brain, heart, and adrenal glands (Cohn et al., 1974).

We have recently reported that 30 min of transient hypoxia of the mother and fetus (caused by reducing the oxygen content of the mother’s inspired gas) results in increased abundance of live bacteria in the fetal brain 24 h after the hypoxic episode (Zarate et al., 2017). Whole genome sequencing of bacteria recovered from the fetal cerebral cortex revealed that the bacteria isolated from the fetal brain were identical to those isolated from the placenta (Zarate et al., 2017). This suggested a route of transfer from placenta to fetal brain with hypoxia. Concomitant with appearance of bacteria in fetal brain is upregulation of molecular pathways that subserve inflammation (Chang et al., 2016a; Zarate et al., 2017). As we have previously reported, the pattern of activation of genes within inflammation-related pathways is consistent with the response to bacterial entry into the fetal brain after hypoxia (Zarate et al., 2017).

We have previously reported that ketamine, an FDA-approved agent used in Neonatal Intensive Care Units (Anand, 2007), attenuated the activation of inflammation-related pathways in fetal cerebral cortex and kidney (Chang et al., 2016a,b). Since the discovery of hypoxia-induced bacterial invasion of the fetus, we have concluded that the increase in abundance of bacteria was causative of the increased inflammation-like response in the fetal brain (Zarate et al., 2017). The mechanism of the effect of ketamine – originally hypothesized to reduce inflammation by blockade of NMDA-mediated glutamate signaling – was therefore unclear (Powers and Wood, 2007; Knutson and Wood, 2010). In this report we test the hypothesis that the effect of ketamine will be reflected by changes in inflammation-related genes in the hypothalamus and hippocampus. Furthermore, we test the hypothesis that ketamine reduces the influx of bacteria into the fetal brain, thereby blocking the stimulus to inflammation. We have reported, from these experiments, upregulated inflammation pathways in cerebral cortex, hypothalamus and hippocampus, as well as kidney cortex in response to hypoxic hypoxia. We have also reported the transfer of bacteria into the brains of these fetuses. This report specifically addresses the effect of ketamine on the inflammation response in hypothalamus and hippocampus, and addresses the question of whether ketamine reduces or prevents the appearance of bacteria in these brain regions.

## Materials and Methods

### Ethics Statement

All experiments were approved by the University of Florida Animal Care and Use Committee and were performed in accordance with the Guiding Principles for Use of Animals of the American Physiological Society.

### Fetal Ovine Surgery

The surgical procedures for fetal and maternal chronic femoral arterial and venous catheterizations were described previously (Chang and Wood, 2015; Chang et al., 2016b). Briefly, ewes were fasted 24 h before surgery, and received 750 mg ampicillin (Polyflex^®^, Boehringer Ingelheim VetMedica, Inc., St. Joseph, MO, United States) before the induction of anesthesia with 0.5–2% isoflurane with oxygen and intubation. The fetal hindlimbs were catheterized with a set of femoral arterial, venous, and amniotic catheters. Ampicillin (500 mg) was injected into the amniotic fluid before the uterus was sutured closed. The ewe also received a set of femoral arterial and venous vascular catheters. In addition, a non-occlusive catheter was implanted in the maternal trachea for the infusion of nitrogen gas as previously described (Chang and Wood, 2015). Post-operative care was provided to the ewe for a minimum of 5 days, including prophylactic administration of ampicillin (15–20 mg/kg, IM, bid), wound care, and monitoring of rectal temperature, food consumption and signs of infection or distress.

### *In vivo* Transient Hypoxia

Studies were performed on chronically catheterized singleton (*n* = 1) and twin (*n* = 15) ovine fetuses at gestational age 122 ± 5 days (full term = 145–147 days), while the ewes were conscious and freestanding in their pens with access to food. Each pregnancy was randomly assigned to the experimental protocol of normoxia or hypoxia, therefore producing the four groups: normoxia control (NC), normoxia+ketamine (NK), HC, and hypoxia+ketamine (HK). For groups treated with ketamine, the fetuses received 3 mg/kg of ketamine intravenously through the femoral venous catheter 10 min prior to normoxic or hypoxic stimuli (30 min). Transient hypoxia was produced by infusing nitrogen gas into the maternal tracheostomy tube, resulting in a 50% decrease in arterial partial pressures of oxygen (P_a_O_2_) for both the ewe and the fetus. Changes in blood gas compositions (ABL80 Radiometer, Copenhagen, Denmark) were closely monitored by anaerobically drawing arterial blood (1 mL) at regular intervals. Fetal physiological responses (hemodynamics, blood gasses, and neuroendocrine values) to hypoxia, ketamine, and the combination of hypoxia and ketamine have been reported previously (Chang and Wood, 2015). Fetuses were euthanized 24 h post initial stimulation of normoxia or transient hypoxia. The fetal brain was hemisected, with one half reserved for fixation and histological analysis and with the other half dissected for molecular analysis. Hypothalamus was removed as a single block of tissue, bounded on the rostral edge by the rostral edge of the optic chiasm, on the caudal side by the caudal edge of the median eminence, and on the side by the edges of the median eminence (Gersting et al., 2009). Hippocampus was dissected as the entire hippocampus (Gersting et al., 2009). Medulla oblongata was defined as the area between the obex and the caudal medulla-rostral spinal cord border (Gersting et al., 2009).

### Microarray Procedures

Total mRNA was extracted from snap frozen fetal hypothalamus tissues using Trizol extraction followed by RNeasy Plus Mini Kit (Qiagen, Valencia, CA, United States), with on-column DNase digestion. RNA integrity number (RIN) values ranged between 8.0 and 9.0, RNA was labeled with cyanine 3 CTP with the Quick Amp Labeling Kit (Cat# 5190-0442, Agilent Technologies, Santa Clara, CA, United States), according to the manufacturer’s protocol. The specific activities of the labeled cRNAs ranged from 11.6 to 15.2 pmol Cy3/μg RNA and yielded 8.9 to 10.96 μg. The cRNA samples were hybridized and processed for one-channel Sheep Gene Expression Microarray (8 × 15 K slide) – 8 arrays with 15208 oligomers each (Cat# G4813A-019921, Agilent Technologies, Santa Clara, CA, United States) as described (Rabaglino et al., 2014). The slides were scanned with Microarray Scanner System (G2505-90021, Agilent) and the measured fluorescence was detected and converted using Agilent Feature Extraction 9.1 software at the Genomics Division of the University of Florida’s Interdisciplinary Center for Biotechnology Research. All microarray data have been uploaded to the Gene Expression Omnibus of the National Center for Biotechnology Information (accession numbers GSE82016 and GSE97916). We performed array analyses in the hippocampus and the hypothalamus for the following groups: NC (*n* = 3), NK (*n* = 4), HC (*n* = 5), and HK (*n* = 4).

### Network Inference and Analysis, and Functional Annotation of Gene Ontology

Gene networks were inferred using the GeneMania (Montojo et al., 2010) plugin of Cytoscape (Shannon et al., 2003). The functional annotation of gene ontology for groups or networks of genes was analyzed using WEB-based GEne SeT AnaLysis Toolkit (WebGestalt) (Zhang et al., 2005; Wang et al., 2013).

### Immunohistochemistry

Fetal brain tissues (*n* = 4 per group) were fixed in 4% buffered paraformaldehyde overnight, and stored in 70% reagent alcohol until embedded in paraffin wax. All tissue samples were sectioned with a microtome (5 μm) and counterstained with methyl green. To identify the presence of microglia or macrophages, the hypothalamus, hippocampus, and medulla were stained for Iba-1 (ionized calcium binding adaptor protein-1) antibody (Cat.# 019-19741, Waco Pure Chemical Industries, Richmond, VA, United States; 1:500 dilution) and peroxidase-PAP sandwich technique (Vectastain, Vector Labs, Burlingame, CA, United States). For each animal, five (hypothalamus and medulla) and ten (hippocampus) images (40× magnification) were randomly selected for quantification. Vascular integrity was assessed using immunostaining of ovine albumin (anti-Sheep Serum Albumin Polyclonal Antibody MBS715170, MyBiosource Inc., San Diego, CA, United States, dilution 1:200) with peroxidase-PAP sandwich visualization. The number of broken and leaky blood vessels were counted using seven (hypothalamus), ten (cerebral cortex), and five (medulla) random images (10× magnification) per animal. All microscopic quantification was done using a blinded protocol.

### Real-Time PCR (qPCR)

The same set of mRNA used for the microarray was also used for qPCR validations. The primers were designed based on the known *Ovis aries* and *Bos taurus* genomes (Table 1) for SYBR green or TaqMan chemistry. Chemistries used for this analysis were Applied Biosystems Fast SYBR Green master mix, and Taqman Fast Advanced master mix (Cat. # 4385612, 4444558, respectively; Thermo-Fisher Scientific, Waltham, MA, United States). The ovine β-actin primers and probe were used as the house keeping gene control, as described previously (Chang et al., 2016b). Bacterial 16S rRNA was measured using universal 16S primers as described by Nadkarni and coworkers (Nadkarni et al., 2002). For each sample, the relative mRNA expression was calculated by the difference in threshold cycle (Δ*C*_t_) between the triplicate mean C_t_ for each gene and for β-actin.

**Table 1:** Primers and probes used for qPCR analysis (all sequences 5′–3′).

Gene	Forward primer	Reverse primer	Probe
ACTB	TTCCTTCCTGGG CATGGA	GACGTCACACTT CATGATGGAATT	TCCTGCGGCA TTCACGAAACT ACCTT
CASP8	TGGCTGCCCTCA AGTTCCT	GGAATAGCATCA AGGCATCCTT	SYBR Green
CD14	CCTAAAGGAC TGCCGACCAA	GCGGCTCCCTG CTTAGCT	SYBR Green
CRH	TCCCATTTC CCTGGATCTCA	GAGCTTGCTGCG CTAACTGA	TTCCACCTCCT CCGAGAAGTCTT GGAAAT
CXCL10	TTGAACTGATTC CTGCAAGTCA	TTCCTTTTCATT GTGGCAATAATCT	SYBR Green
IL1B	CGTGGCCAT GGAGAAGCT	GGTCATCATCACG GAAGACATGT	SYBR Green
MYD88	GCCTGAGTATTT TGATGCCTTCA	GCTGCCGGATC ATCTCATG	SYBR Green
NFKB1	TCCCACAGATGT TCACAAACAGT	GACGCTCAATCTT CATCTTGTGAT	SYBR Green
NFKBIA	CTACACCTTGCC TGTGAGCA	AGACACGTGTGG CCATTGTA	SYBR Green
OAS1	GAGGAAAGAGG GCGAGTTCT	GGATGAGGCTCT TCAGCTTG	SYBR Green
PTGS2	GCACAAATCTGA TGTTTGCATTCT	CTGGTCCTCGTT CATATCTGCTT	TGCCCAGCACTT CACCCATCAATTTT
PTX3	GCACCTGGGATT CAAAGAAA	TGTTTCATCAAA GCCACCAA	SYBR Green
TLR2	GATTCTGCTGGA GCCCATTG	TCATGATCTTCCG CAGCTTACA	SYBR Green
TLR4	ACTCGCTCCGG ATCCTAGACT	CCTTGGCAAATT CCGTAGTTCT	SYBR Green
TNF	CCCTTCCACCCC CTTGTT	ATGTTGACCTTG GTCTGGTAGGA	SYBR Green

### Statistics

As previously described, statistical analysis for Agilent 15.5 k array was performed with Bioconductor’s Limma package for R software v.2.15.1, which utilized moderated *t*-test and empirical Bayes method for small sample size per group (*p* < 0.05) (Chang et al., 2016b). The qPCR data were analyzed by Student’s *t*-test (Zar, 1984). Two-way nested ANOVA design was applied for immunohistochemistry data analysis, using the Genmod Procedure of SAS/STAT^®^ 9.3 (SAS Institute Inc., Cary, NC, United States). Data are presented as mean values ± standard error of the mean (SEM), and the criterion for statistical significance was *p* < 0.05.

## Results

The blood gas and cardiovascular responses to hypoxia and normoxia, and the effect of ketamine on these variables in a larger cohort of experiments have been previously reported (Chang and Wood, 2015).

Twenty-four hours after the onset of a 30 min period of hypoxia, 280 genes were upregulated and 357 genes were downregulated in hypothalamus compared to fetuses subjected to normoxia alone. In hippocampus, 270 genes were upregulated and 240 were downregulated. We have previously reported these changes in gene expression as well as statistically significant over-represented gene ontology terms and KEGG pathways activation for these differentially regulated genes elsewhere (Zarate et al., 2017). Inflammation- and infection-related pathways were significantly upregulated in both brain regions (Zarate et al., 2017).

Ketamine alone (in normoxic fetuses) upregulated 15 genes and downregulated 21 genes in hypothalamus and upregulated 22 and downregulated 29 genes in hippocampus. Gene ontology analysis of the upregulated genes revealed significant association with extracellular matrix binding and collagen binding in hypothalamus, but the downregulated genes did not reveal any significant biological processes in hypothalamus. In the hippocampus, there was no significant biological processes revealed by gene ontology analysis that were associated with either the upregulated or the downregulated genes.

**FIGURE 1:**
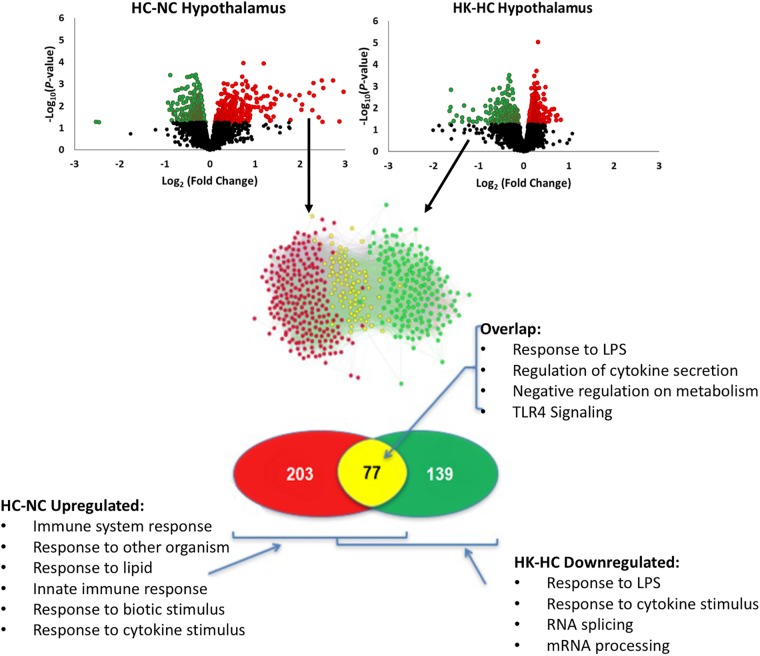
As shown in top panels, analysis of gene expression using Agilent ovine 15.5 k array revealed significant up- and down-regulation of gene expression by hypoxia compared to normoxia [HC-NC, previously reported (Zarate et al., 2017)], as well as significant up- and down-regulation of gene expression in hypoxic animals by ketamine (HK-HC). Network inference and statistical modeling of gene ontology terms revealed that hypoxia upregulated pathways related to inflammation, and that ketamine was effective at decreasing the gene expression in those pathways in the hypoxic animals. Transcriptomic modeling indicated a high likelihood of the entire inflammation pathway from TLR2 and TLR4 being involved in the response. HC-NC plots were previously published under a Creative Commons Attribution 4.0 International License in Zarate et al. (2017).

**FIGURE 2:**
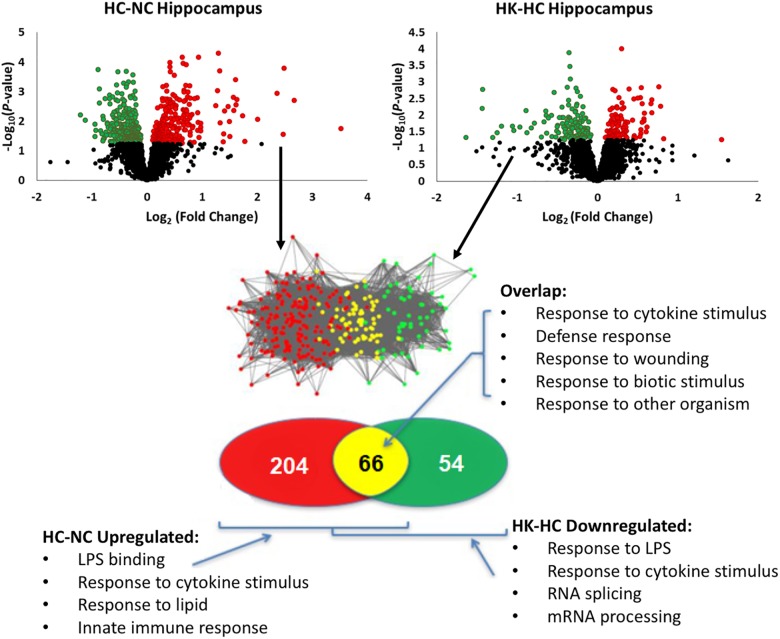
Top panels, significant up- and down-regulation of gene expression in hippocampus by hypoxia compared to normoxia [HC-NC, previously reported (Zarate et al., 2017)], as well as significant up- and down-regulation of gene expression in hypoxic animals by ketamine (HK-HC). Network inference and statistical modeling of gene ontology terms were as described in legend to Figure 1. HC-NC plots were previously published under a Creative Commons Attribution 4.0 International License in Zarate et al. (2017).

Ketamine had a substantial effect on the transcriptomic response to hypoxia. In hypothalamus, ketamine decreased the expression of 293 genes in and increased the expression of 238 genes in the fetuses subjected to hypoxia (Figure 1). In hippocampus, ketamine upregulated 125 and downregulated 130 genes when compared to hypoxia alone (Figure 2).

Comparison of the genes upregulated by hypoxia but downregulated by ketamine after hypoxia, allows identification of those pathways that are activated by hypoxia but whose activation is reversed by ketamine pretreatment. This analysis in hypothalamus revealed 77 genes with this pattern (Figure 1). Gene ontology analysis revealed that these genes were significantly associated with immune response, cellular response to lipopolysaccharide, response to other organism, innate immune response, and response to molecule of bacterial origin (Table 2). KEGG pathways significantly associated with these genes include MAPK signaling pathway, cytokine-cytokine receptor interaction, hematopoietic cell lineage, chemokine signaling, and TLR signaling pathway (Table 2).

**Table 2:** Top 10 gene ontology biological processes and enriched KEGG pathways associated with genes that were significantly up regulated in the hypothalamus during acute hypoxic stress, but were down regulated with ketamine.

Analysis	Pathways, processes, functions, components	# Genes involved	Adjusted *P*-values
Biological	Defense response	30	1.93E-11
Process	Response to stress	45	2.09E-10
	Immune response	26	7.72E-09
	Immune system process	33	7.72E-09
	Regulation of defense response	16	1.67E-07
	Response to lipopolysaccharide	12	2.05E-07
	Response to other organism	18	2.05E-07
	Response to wounding	24	2.30E-07
	Innate immune response	17	2.85E-07
	Response to molecule of bacterial origin	12	2.85E-07
KEGG	MAPK signaling pathway	9	1.05E-07
Pathways	Osteoclast differentiation	7	1.17E-07
	Cytokine-cytokine receptor interaction	8	6.45E-07
	Hematopoietic cell lineage	5	8.36E-06
	Toll-like receptor signaling pathway	5	1.28E-05
	Chemokine signaling pathway	6	1.28E-05
	Pathways in cancer	7	1.94E-05
	Toxoplasmosis	5	2.96E-05
	Hepatitis C	5	3.96E-05
	Adipokine signaling pathway	4	4.60E-05

The same analysis strategy in hippocampus revealed 66 genes that were upregulated by hypoxia but downregulated by ketamine (Figure 2). Significantly associated biological process terms of these genes included response to cytokine stimulus, response to lipopolysaccharide, response to molecule of bacterial origin, response to external stimulus, and response to stress (Table 3). KEGG pathways significantly overrepresented in this gene set were cytokine-cytokine receptor interaction, TLR signaling pathway, MAPK signaling pathway, adipocytokine signaling pathway, chemokine signaling pathway, osteoclast differentiation, and JAK/STAT signaling pathway (Table 3).

**Table 3:** Top 10 gene ontology biological processes and enriched KEGG pathways associated with genes that were significantly up regulated in the hippocampus during acute hypoxic stress, but were down regulated with ketamine.

Analysis	Pathways, processes, functions, components	# Genes involved	Adjusted *P*-values
Biological	Response to lipid	18	4.49E-08
Process	Response to cytokine stimulus	15	1.49E-06
	Response to organic substance	27	1.49E-06
	Response to biotic stimulus	16	1.53E-06
	Response to chemical stimulus	33	1.53E-06
	Response to other organism	15	4.53E-06
	Response to lipopolysaccharide	10	4.53E-06
	Response to molecule of bacterial origin	10	6.95E-06
	Response to external stimulus	21	1.54E-05
	Response to stress	32	1.61E-05
KEGG Pathways	Cytokine-cytokine receptor interaction	7	8.15E-06
	Malaria	4	2.32E-05
	Toll-like receptor signaling pathway	4	2.00E-04
	MAPK signaling pathway	5	6.00E-04
	Adipocytokine signaling pathway	3	1.20E-03
	Chemokine signaling pathway	4	1.50E-03
	Amoebiasis	3	3.10E-03
	Osteoclast differentiation	3	4.60E-03
	African trypanosomiasis	2	5.20E-03
	Jak-STAT signaling pathway	3	6.50E-03

There were 57 genes in hypothalamus and 31 in hippocampus that were downregulated by hypoxia but increased in the fetuses subjected to hypoxia and ketamine. In both brain regions, these genes were not significantly associated with gene ontology terms. KEGG analysis of the hypothalamic genes revealed a significant association with metabolic pathways, vitamin digestion and absorption, fructose/mannose metabolism, endocytosis, and spliceosome, while a similar analysis in hippocampus did not yield any significantly associated KEGG pathways.

**FIGURE 3:**
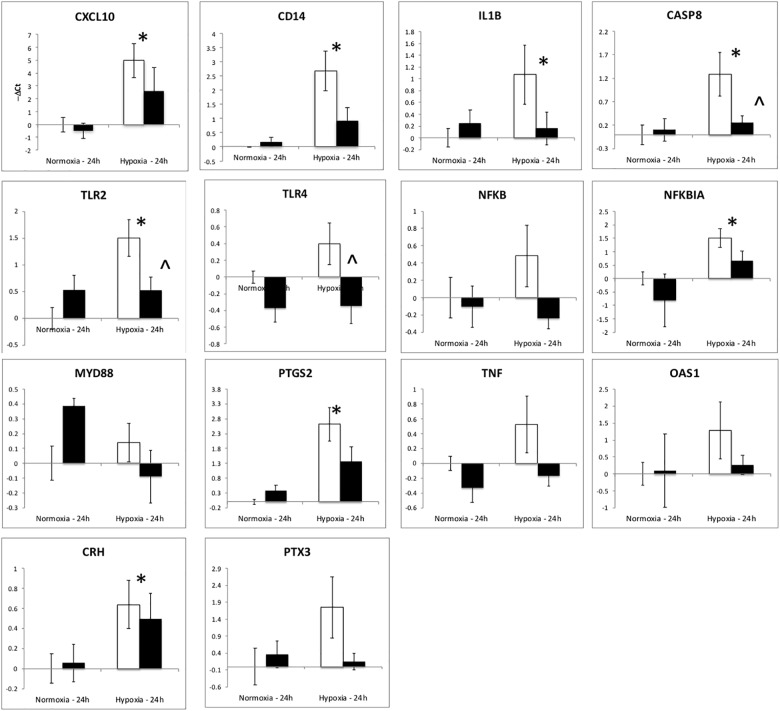
Gene expression (mRNA abundance) in fetal hypothalamus measured by real-time qPCR for genes in the toll-like receptor inflammation pathway. Values are represented as negative delta cycle threshold (–Δ*C*_t_) compared to the NC group. Open bars represent experiments in which ketamine was not administered. Filled bars represent experiments in which ketamine was administered before normoxia or hypoxia. The criterion for statistical significance was *P* < 0.05 (Student’s *t*-test). Data are presented as means ± SEM, and the y-axis scale varies between plots. ^∗^Statistically significant difference of hypoxia group compared to normoxia control. ∧Statistically significant difference of hypoxia control group compared to hypoxia + ketamine group.

**FIGURE 4:**
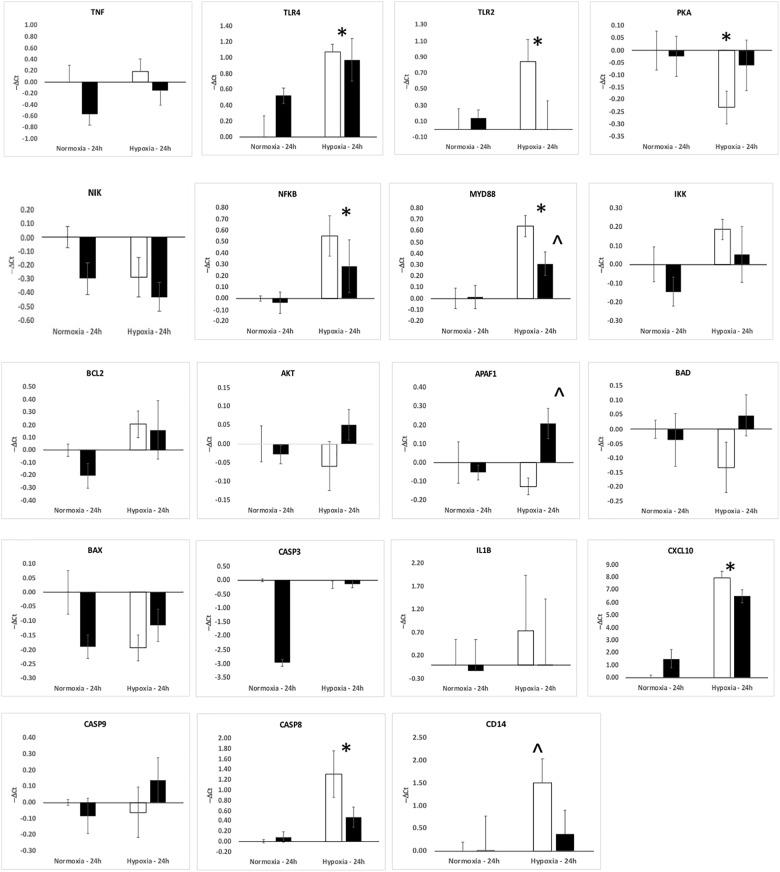
Gene expression (mRNA abundance) in fetal hippocampus measured by real-time qPCR for genes in the toll-like receptor inflammation pathway. Values are represented as negative delta cycle threshold (–Δ*C*_t_) compared to the NC group. Open bars represent experiments in which ketamine was not administered. Filled bars represent experiments in which ketamine was administered before normoxia or hypoxia. The criterion for statistical significance was *P* < 0.05 (Student’s *t*-test). Data are presented as means ± SEM, and the y-axis scale varies between plots. ^∗^Statistically significant difference of hypoxia group compared to normoxia control. ∧Statistically significant difference of hypoxia control group compared to hypoxia + ketamine group.

The upregulation of inflammation-related genes (MYD88, NFKB, etc.) and the subsequent modeling of the transcriptome led us to further explore the relationship between hypoxia and inflammation, and the modification of this relationship by ketamine at both the RNA and histological levels. We chose to probe, at the mRNA expression level, the TLR pathway and the apoptosis pathway. Hypoxia increased the abundance of mRNA for multiple inflammation-related genes in hypothalamus (Figure 3) and hippocampus (Figure 4), including TLR2, CD14, NFKBIA, IL1B, CXCL10, PTGS2, and CASP8 (previously reported in Zarate et al., 2017). Ketamine reduced the CD14, TLR2, TLR4, MYD88, NFKB, and CASP8 mRNA abundance in one or both of these brain regions compared to fetuses that were subjected to hypoxia but not to ketamine treatment (Figures 3, 4).

**FIGURE 5:**
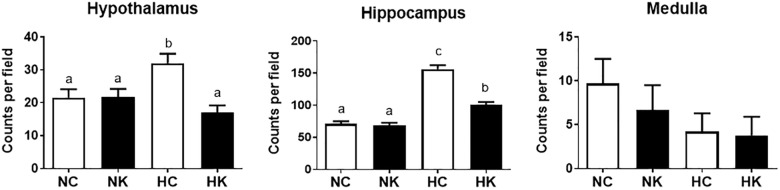
Ketamine reduces the number of microglia and macrophages in the hypothalamus and hippocampus 24 h after transient hypoxia. For each brain region, the Iba-1 positive cells counted per 40X field (average of 7 fields analyzed for each animal) were averaged from 4 animals per group. Data from hippocampus are average measurements from regions CA1, CA2, CA3, CA4, and Dentate Gyrus. Data are expressed as mean ± SEM. Different letters indicate statistically significant difference (*P* < 0.05). NC, normoxia control; NK, normoxia + ketamine; HC, hypoxia control; HK, hypoxia + ketamine.

The gene ontology analysis and the qPCR analysis indicated that a major component of the response 24 h after hypoxia is inflammation, and more specifically genes related to the toll-like receptor (TLR2 and TLR4) inflammation pathway. We have reported, previously, that hypoxia increases microglia/macrophage abundance in cerebral cortex (Chang et al., 2016a), renal cortex (Chang et al., 2016b), and in hypothalamus and hippocampus (Zarate et al., 2017). We have also previously reported that anti-inflammatory actions of ketamine pretreatment were accompanied by reductions in microglia/macrophages in both fetal cerebral (Chang et al., 2016a) and renal (Chang et al., 2016b) cortex. In this report, we extend this effect of ketamine to hypothalamus and hippocampus (Figure 5).

**FIGURE 6:**
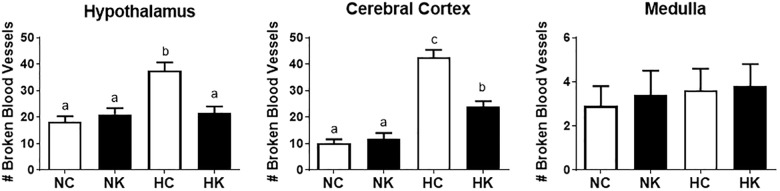
Ketamine reduces the number of broken or leaky blood vessels in the fetal hypothalamus and cerebral cortex 24 h after transient hypoxia. Number of broken blood vessels were counted in 10X field images and averaged for each animal per brain region: hypothalamus, cerebral cortex, and medulla. Data are expressed as means ± SEM (*n* = 4/group). Different letters indicate statistically significant difference (*P* < 0.05). NC, normoxia control; NK, normoxia + ketamine; HC, hypoxia control; HK, hypoxia + ketamine.

**FIGURE 7:**
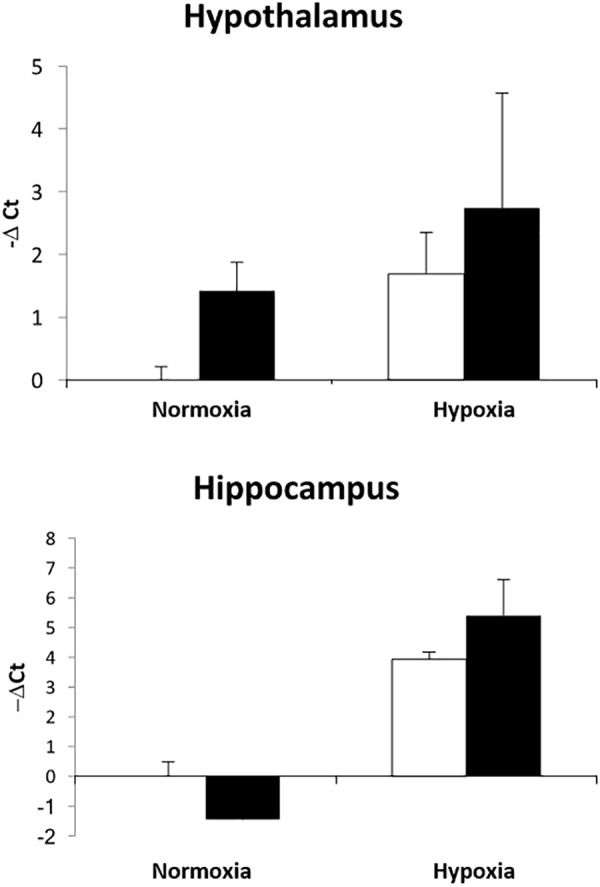
Effect of ketamine on the expression of 16S bacterial rRNA in hypothalamus and hippocampus 24 h after transient hypoxia. Data are presented as –Δ*C*_t_ values relative to the NC group for each brain region (*n* = 4 animals per group). Data are expressed as mean ± SEM.

We have previously demonstrated that hypoxia resulted in an increase in the abundance of bacterial 16S rRNA gene expression and vascular permeability in fetal cerebral cortex, hypothalamus, and hippocampus (Zarate et al., 2017). Ketamine significantly reduced the effect of hypoxia on vascular integrity in hypothalamus and cerebral cortex (Figure 6) and decreased the macrophage/microglia infiltration of hypothalamus and hippocampus (Figure 5). Likewise, ventilatory hypoxic hypoxia produced an increase in bacterial load though an upregulation of 16S gene; however, ketamine did not significantly reduce or block the hypoxia-induced increase in qPCR-detectable 16S bacterial rRNA (Figure 7). While not specifically the topic of this analysis, medulla was analyzed for Iba-1 and albumin immunostaining: neither hypoxia nor ketamine had any effect on either of these measured variables in medulla (Figures 5, 6).

## Discussion

We hypothesized that ketamine would reduce hypothalamic inflammation secondary to its action to block NMDA receptor activity, thereby reducing the damaging effects of intense glutamate neurotransmission. Our original concept was that glutamate neurotransmission, followed by calcium influx into neurons, would increase apoptosis and cell death, ultimately initiating inflammation. Calcium is also released from macrophages into the extracellular space. It is possible that cell death and the associated release of cellular contents into the extracellular space could cause or contribute to the inflammation and increase in macrophage/microglia in hypothalamus and hippocampus. While the results of the present study do not reveal the ultimate cause of the inflammation, they do demonstrate that the entire inflammation cascade at a gene transcript level is activated, similar to the response to infection. Ketamine attenuates the activation of genes in inflammation-related pathways.

Although, we designed this experiment on the assumption that the mechanism of action of ketamine was the blockade of NMDA receptors, we propose that the anti-inflammatory action of the drug in the context of the present experiments is likely to involve other mechanisms. For example, the results could be explained by an action of ketamine to block macrophage activation or translocation. Shimaoka and colleagues reported that ketamine, but not other NMDA receptor antagonists, reduced nitrate production and TNFα production by J774 cells (a murine macrophage-like cell line) (Shimaoka et al., 1996). Ketamine reduces NFkB activation in A172 human glioblastoma cells exposed to LPS (Sakai et al., 2000) and reduces TNFα and IL6 production by LPS-stimulated RAW 267.4 (transformed mouse macrophage) cells (Wu et al., 2008). The mechanism of action may involve a reduction in ERK1/2 phosphorylation (Chang et al., 2010) and may, in cases of gram negative bacterial infection, interfere with LPS binding protein binding to LPS (Chen et al., 2009). The majority of bacteria appearing in fetal brain in the present experiments are gram positive (*Staphylococcus*), as reported previously (Zarate et al., 2017). Ketamine is known to reduce TLR2-mediated activation of ERK1/2 and NFKB by lipoteichoic acid, the principal component of cell wall in gram positive bacteria (Chang et al., 2010). However, in this study, ketamine did not produce any significant effects on bacterial load in the fetal hippocampus or hypothalamus. We believe that ketamine failed to have any affects on bacterial 16S gene expression because it has neither antibiotic properties nor any obstructive effects on the bacterial movement from maternal to fetal circulation. Histological data also indicates that hypoxia negatively affects the integrity of the blood vessels, and ketamine administration reduced the number of broken vessels. We have previously reported (Chang and Wood, 2015) that ventilatory hypoxia produces hemodynamic modifications in the fetus, and this response was attenuated by ketamine. Alterations in hemodynamics during acute episodes of hypoxia have been reported to affect the microvascular system, especially in the brain (Mark and Davis, 2002; Luo et al., 2012; Baburamani et al., 2013). These previous findings strongly supports our data and together they provide new insights on the therapeutical effects of ketamine in episodes of hypoxia-induced cerebral edema (Shu et al., 2012). The exact mehanisms on how blood vessels integrity are affected by hypoxia are not fully elucidated.

It is perhaps tempting to assume that inflammation in the fetal hypothalamus is damaging, or that inflammation might “program” an increased propensity for adult disease. The results of the present study do not, however, address the question of whether inflammation and the increase in tissue macrophages/microglia is damaging or whether the changes that we observe are reparative. Indeed, it is not clear from the present experiments that the inflammation reflects damage or cell death in any of the brain regions. Microglia and macrophages are important participants in tissue development and maturation. For example, microglia appear to be important in the turnover of neuronal progenitor cells in the developing brain. Arno and colleagues have proposed that microglia are a critical part of maintaining the balance between cell division and cell death in the developing brain (Arno et al., 2014). Loss of microglial function might be causative with regard to known conditions involving cellular overgrowth (Arno et al., 2014). Unknown variables important to fetal brain inflammation may have great relevance to the ultimate outcome of the offspring. Knowing, for example, that fetal brain response to transient hypoxic hypoxia involves several heretofore unrecognized variables – including bacteria after, for example, maternal antibiotic treatment or prophylactic indomethacin treatment during pregnancy or labor/delivery, or the use of ketamine for analgesia in the Neonatal Intensive Care Unit.

We conclude that the action of ketamine in the fetal brain (or, perhaps in the premature neonatal brain when ketamine is used in the Neonatal Intensive Care Unit) reflects a reduction in the inflammatory response to bacteria or to other pathogen-associated molecular patterns to which the brain is exposed. Future work should perhaps be focused on the programming effects of maternal ventilatory hypoxia as well as elucidating possible sex effects differences induced by hypoxic episodes in late gestation.

## Author Contributions

EC and CW conceived and designed the experiments, collected, analyzed, and interpreted the data, drafted the article, and critically revised the article for important intellectual content. MZ, TA, and MR collected, analyzed and interpreted the data, and critically revised the article for important intellectual content. ER and MK-W interpreted the data and critically revised article for important intellectual content. All authors approved the final version submitted for publication.

## Conflict of Interest Statement

The authors declare that the research was conducted in the absence of any commercial or financial relationships that could be construed as a potential conflict of interest.

## Abbreviations

CASP8: caspase8
CD14: CD14 molecule
CXCL10: chemokine (C-X-C motif) ligand 10
ERK: extracellular signal-regulated kinase
HC: hypoxic control
HK: hypoxia + ketamine
IL1β: interleukin 1 beta
JAK/STAT: janus kinase/signal transducers and activators of transcription
KEGG: Kyoto Encyclopedia of Genes and Genomes
LPS: lipopolysaccharides
MAPK: mitogen-activated protein kinases
MYD88: myeloid differentiation primary response 88
NC: normoxic control
NFκB: nuclear factor kappa-light-chain-enhancer of activated B cells
NK: normoxia + ketamine
NMDA: *N*-Methyl-_D_-aspartate
PTGS2: prostaglandin-endoperoxide synthase 2
TLR: toll-like receptor
TNFα: tumor necrosis factor-alpha
